# Effects of Carfilzomib Therapy on Left Ventricular Function in Multiple Myeloma Patients

**DOI:** 10.3389/fcvm.2021.645678

**Published:** 2021-04-21

**Authors:** Giulia Mingrone, Anna Astarita, Lorenzo Airale, Ilaria Maffei, Marco Cesareo, Teresa Crea, Giulia Bruno, Dario Leone, Eleonora Avenatti, Cinzia Catarinella, Marco Salvini, Giusy Cetani, Francesca Gay, Sara Bringhen, Franco Veglio, Fabrizio Vallelonga, Alberto Milan

**Affiliations:** ^1^Department of Internal Medicine and Hypertension Division, “Città della Salute e della Scienza” Hospital, University of Turin, Turin, Italy; ^2^Myeloma Unit, Division of Haematology, “Città della Salute e della Scienza” Hospital, University of Turin, Turin, Italy

**Keywords:** cardio-oncology, echocardiography, global longitudinal strain, arterial hypertension, cardiovascular organ damage, multiple myeloma

## Abstract

**Background:** Carfilzomib improves the prognosis of multiple myeloma (MM) patients but significantly increases cardiovascular toxicity. The timing and effect of Carfilzomib therapy on the left ventricular function is still under investigation. We sought to assess the echocardiographic systo-diastolic changes, including global longitudinal strain (GLS), in patients treated with Carfilzomib and to identify predictors of increased risk of cardiovascular adverse events (CVAEs) during therapy.

**Methods:** Eighty-eight patients with MM performed a baseline cardiovascular evaluation comprehensive of transthoracic echocardiogram (TTE) before the start of Carfilzomib therapy and after 6 months. All patients were clinically followed up to early identify the occurrence of CVAEs during the whole therapy duration.

**Results:** After Carfilzomib treatment, mean GLS slightly decreased (−22.2% ± 2.6 vs. −21.3% ± 2.5; *p* < 0.001). Fifty-eight percent of patients experienced CVAEs during therapy: 71% of them had uncontrolled hypertension, and 29% had major CVAEs or CV events not related to arterial hypertension. GLS variation during therapy was not related to an increased risk of CVAEs; however, patients with baseline GLS ≥ −21% and/or left ventricular ejection fraction (LVEF) ≤ 60% had a greater risk of major CVAEs (OR = 6.2, *p* = 0.004; OR = 3.7, *p* = 0.04, respectively). Carfilzomib led to a higher risk of diastolic dysfunction (5.6 vs. 13.4%, *p* = 0.04) and to a rise in E/e′ ratio (8.9 ± 2.7 vs. 9.7 ± 3.7; *p* = 0.006).

**Conclusion:** Carfilzomib leads to early LV function impairment early demonstrated by GLS changes and diastolic dysfunction. Baseline echocardiographic parameters, especially GLS and LVEF, might improve cardiovascular risk stratification before treatment.

## Introduction

Carfilzomib is a second-generation irreversible proteasome inhibitor (PI) approved for the treatment of relapsed or refractory multiple myeloma (RRMM) (1, 2). Its efficacy has been established in advanced MM, but the antiproteasome activity is burdened by cardiovascular (CV) adverse effects, such as arterial hypertension, arrhythmia, new-onset or worsening heart failure, dyspnea, coronary heart disease, venous thromboembolism, renal failure, pulmonary hypertension, and cardiac-related sudden death (2–11). Despite these known effects, the exact mechanism and early marker of toxicity of Carfilzomib on cardiac structure and function have not been established. The PI seem to be toxic to cardiomyocytes probably because of the apoptosis caused by accumulation of unfolded, damaged proteins due to proteasome inhibition. In addition, Carfilzomib may induce endothelial dysfunction altering endothelial nitric oxide synthase activity and nitric oxide levels (12–17).

Guidelines and expert position statements (18–20) recommend baseline CV risk assessment for patients scheduled to receive potentially cardiotoxic therapies, and different approaches for early detection and prevention of CV diseases have been developing (21–23). The prognostic and predictive role of echocardiographic monitoring in patients undergoing treatment is still under investigation.

Left ventricle ejection fraction (LVEF) has been the most widely used echocardiographic parameter to evaluate cardiotoxicity related to cancer treatments; however, its sensibility in detecting minimal contractility variations is low. Global longitudinal strain (GLS), assessed using 2D speckle-tracking echocardiography (2D-STE), is a recommended technique for detecting and quantifying early disturbances in LV systolic function (19, 24–26). The GLS prognostic value in predicting cardiovascular events in general population and its association with all-cause mortality in patients receiving chemotherapy has already been demonstrated (27, 28).

Therefore, the aim of our study is to assess echocardiographic systolic and diastolic changes, including GLS modifications, in patients treated with Carfilzomib, in order to verify their potential predictive value on new incidence of cardiovascular adverse events (CVAEs) during therapy.

## Methods

### Study Design

From January 2015 to March 2020, 116 MM patients followed by the Myeloma Unit (“Città della Salute e della Scienza,” Turin) underwent a baseline CV evaluation before Carfilzomib treatment; 88 of them completed the 6 months echocardiographic follow-up (FU) examination and were enrolled. Subsequently, all patients were clinically followed for the duration of chemotherapy to assess the incidence of CVAEs. To be included in the study, patient had to be >18 years old and have a diagnosis of MM with clinical indication to Carfilzomib treatment. Patients were excluded in the presence of cardiac amyloidosis or poor-quality echocardiographic scans.

The study protocol was approved by the ethic committee of our hospital “A.O.U. Città della Salute e della Scienza” of Turin (Protocol Number 0038655), and each patient signed a written consent form.

**Figure 1:**
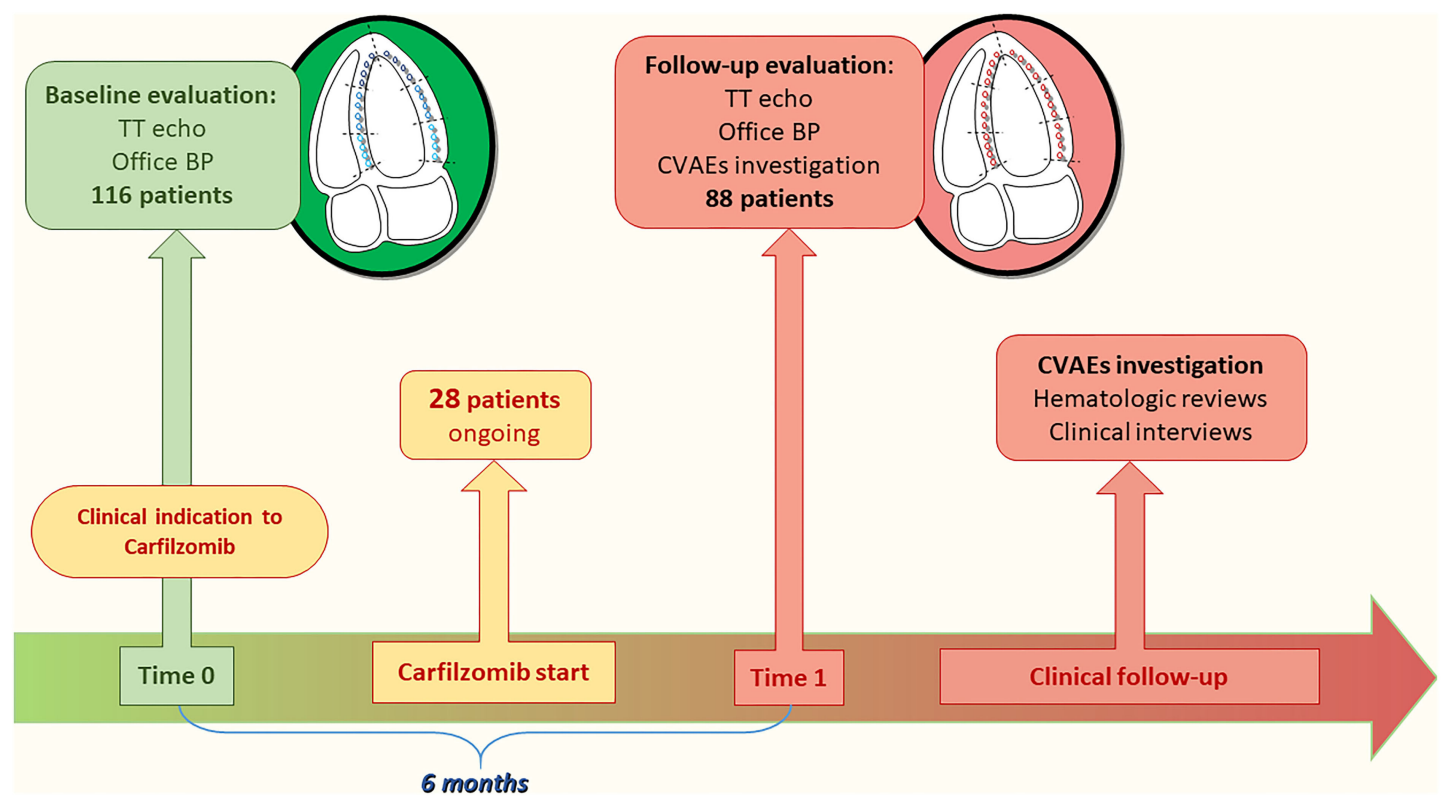
Population and study design. TT, transthoracic; BP, blood pressure; CVAEs, cardiovascular adverse events.

Patients underwent a comprehensive cardiovascular evaluation at our EchoLab (Hypertension Unit, University of Turin) before the beginning of Carfilzomib infusions and after 6 months of therapy. The evaluation consisted of clinical-anamnestic assessment, office blood pressure (BP) measurement, electrocardiogram, and transthoracic echocardiography (TTE). Subsequently, all patients were clinically followed up during therapy through periodic review of hematological reports or telephonic interview with a standardized questionnaire aimed at the early identification of CVAEs (Figure 1).

### Blood Pressure Measurement

Office BP measurements were performed according to the current guidelines (29). An automatic sphygmomanometer was used (Omron, M10-IT model). Three BP measurements were performed 1–2 min apart, and the mean value was used for subsequent analysis. Optimal office BP control was defined as the average BP <140/90 mmHg. In case of uncontrolled BP values at baseline, antihypertensive treatment was started or optimized.

### Echocardiography and 2D Speckle Tracking

2D TTE and speckle-tracking analysis were performed at baseline (before Carfilzomib therapy initiation) and after 6 months of therapy following the current guidelines (30).

The TTE was performed at rest with the patient lying on the left lateral decubitus position. Standard 2D images were acquired with an iE33 ultrasound machine (Philips Medical System, Andover, MA, USA) equipped with a sector probe (S5-1 transducer). The images were recorded digitally and analyzed offline by a single operator.

LV diameters and wall thickness were measured in parasternal long-axis view. LV geometry was defined by calculating LV mass (LVM, obtained using the Deveraux formula indexed to body surface area) and relative wall thickness (RWT, obtained dividing the double of the LV inferolateral wall thickness by the LV internal diameter at end diastole). LV diastolic function was defined through the evaluation of early diastolic tissue Doppler (TDI) velocities (e′ waves) of septal and lateral mitral annulus, tricuspid regurgitation peak velocity, left atrial volume indexed to body surface area (LAVi), and E/e′ratio. LV diastolic dysfunction can be diagnosed if more than half of the available parameters are abnormal based on the current cutoffs (31). Mitral valve inflow (E and A wave velocity) and deceleration time are traditionally used to identify the filling patterns, according to the current recommendations (31).

STE analysis, including LVEF assessment, was performed with a commercially available software (Automated Cardiac Motion Quantification, QLAB Cardiac Analysis, Philips, Andover, MA, USA). GLS and LVEF were computed offline from standard 2D images of the LV in apical views (4-/2-chambers for LVEF, 4-/3-/2-chambers for GLS). The workflow for this analysis requires the endocardium border to be traced semiautomatically after identification of three reference points (apex and mitral annulus), with manual adjustment when needed, following standardized protocols (32). At the end of the GLS analysis, the software output consists of global quantification of strain values (expressed as a %, with more negative values indicating greater deformation), and a “bull's eye” map representing the global and regional LV deformation. LVEF was computed with a semiautomatically Simpson biplane method, based on the 4-/2-chamber volumes. Reproducibility of LVEF and GLS assessments was determined with comparisons of 10 double-blinded measurements, obtained by two expert operators (33).

### Cardiovascular Adverse Events Definition

The incidence of CVAEs was detected both at the 6-month cardiovascular evaluation and later through periodic review of patient hematological reports or phone calls during the whole therapy duration. If severe CVAEs occurred before the planned 6 months FU, the planned repeated TTE examination was anticipated.

CVAEs were assessed and graded according to the Common Terminology Criteria for Adverse Events version 5.0 (34). We divided the CVAEs in arterial-hypertension-related events and non-hypertension related (or “major”). Among the former, we included new onset or worsening arterial hypertension, defined as increased BP values (≥140/90 mmHg) requiring additional antihypertensive treatments, BP rises occurred just before or immediately after the Carfilzomib infusion (within 30 min), uncontrolled hypertension (>180/110 mmHg) with related symptoms and without organ damage, hypertensive emergency (symptomatic BP > 180/110 mmHg with target acute organ damage caused). Major CVAEs included dyspnea related to Carfilzomib infusions (within 3 days of infusion), arrhythmias (such as atrial fibrillation, atrial bigeminy, ventricular tachycardia, ventricular bigeminy/trigeminy), severe hypotensive events (≤90/60 mmHg), syncope, cardiac failure, typical chest pain with subsequent negative cardiologic investigations, myocardial infarction, and cardiac arrest.

Based on CVAEs incidence during Carfilzomib treatment, we considered three subgroups: patients who experienced (1) major CVAEs, (2) only arterial-hypertension-related events, and (3) did not experience any adverse event. Patients who experienced both major and arterial-hypertension-related CVAEs were included in group 1 considering the greater clinical implications of major CVAEs.

### Statistical Analysis

Statistical analysis was performed by using SPSS program (IBM SPSS Statistics, Version 22.0.0.0, IBM Corp., Armonk, NY, USA). Quantitative variables were expressed as mean values and standard deviations or median values and interquartile ranges, according to their distribution. Qualitative variables were expressed as absolute values and percentages. Paired Student's *t* test or Wilcoxon test were performed for comparisons before and during/after therapy for quantitative variables, as appropriate, while McNemar test was used for qualitative variables. ANOVA (or non-parametric ANOVA) test was performed to compare quantitative variables between groups; Chi-square test was used for qualitative variables. Logistic regression was utilized to assess the association between baseline echocardiographic parameters and CVAEs risk. A *p* < 0.05 was assumed as level of statistical significance for all analysis.

“R A Language And Environment For Statistical Computing” software (v4.0.0 for Mac OSX, R Core Team; Vienna, Austria) was utilized to calculate interclass coefficient correlation (ICC) estimates with their 95% confident intervals (CIs) (based on a single-rater *k* = 2, absolute-agreement, two-way mixed-effects model) and DeLong test used for receiver operating characteristic (ROC) curves comparison.

## Results

A total of 88 patients met the inclusion criteria and represented our study population. Mean age was 65.4 years, with an equal distribution between genders. Half of the patients had history of hypertension, 11% had diabetes mellitus, and about 16% dyslipidemia. Median MM duration was 4.6 (2.4–7.1) years (Table 1).

**Table 1:** Population characteristics at baseline.

**General characteristics**	**Population, *n* = 88**
Age, years	65.4 ± 8.7
Male sex, *n*(%)	46 (52.3)
Weight, kg	71.8 ± 15
Height, cm	161.7 ± 10.7
BSA, m^2^	1.75 ± 0.2
BMI, kg/m^2^	27.3 ± 4.4
**Cardiovascular risk factors**	
Active smoke, *n*(%)	6 (6.8)
Arterial hypertension, *n*(%)	44 (50)
Obesity, *n*(%)	26 (29.5)
Coronary heart disease, *n*(%)	3 (3.4)
Diabetes mellitus, *n*(%)	10 (11.4)
Chronic renal failure, *n*(%)	9 (10.2)
Dyslipidemia, *n*(%)	14 (15.9)
**Oncological history**	
MM duration, years	4.6 [2.4–7.1]
Previous therapy^*^:	
Anthracyclines, *n*(%)	26 (29.5)
Alkylating agents, *n*(%)	68 (77.3)
Immunomodulating agents, *n*(%)	62 (70.5)
Bortezomib, *n*(%)	76 (86.4)
Auto-transplantation, *n*(%)	63 (71.6)
Relapsed/Refractory MM, *n*(%)	84 (95.4)

* *Patients were mostly treated with multiple therapies; hence, tot % will amount to >100*.

*BSA, body surface area; BMI, body mass index; MM, multiple myeloma*.

### Timing and Dose of Carfilzomib-Based Treatments

Carfilzomib was administered in association with dexamethasone only (31% of patients) or immunomodulant drugs plus dexamethasone (67% of patients) at a standard dose as International Guideline of MM (1) recommended (Supplementary Table 1). Carfilzomib infusions were continued for a median of 10.4 (6.7–18.7) months, the median cumulative dose was 3193 (1494.1–5319.3) mg (at the end of planned therapy or at the time of the study if treatment is ongoing, Supplementary Table 2).

### Hemodynamic and Echocardiographic Modifications After Carfilzomib

Follow-up (FU) cardiovascular evaluations were planned after 6 months of therapy and anticipated, compared to the study plan, whenever a significant event occurred (median, 5.4; interquartile, 4.3–6.4 months). The average cumulative Carfilzomib dose administered until the FU examination was 1413.4 (1140.4–2105.2) mg. All 88 patients underwent a FU TTE; however, due to poor quality of the apical imaging window, GLS and LVEF analysis was performed on 76 patients (86.3% of the population) at baseline and 70 (79.5%) at FU evaluation. Patients in which functional analysis was not possible did not differ in mean age, gender, physical characteristics, and CV risk factors from the others. ICC results demonstrated an excellent reproducibility for GLS (ICC results and CI ≥90%) and a good reproducibility for LVEF assessments (ICC and CI ≥75%, Supplementary Table 3) (33).

Office BP values were reduced at the FU examination after Carfilzomib, with a greater percentage of BP values ≤ 140/90 mmHg. Forty patients (45.5% of the 88 patients) modified the antihypertensive drug after the baseline visit; 14 (35%) of these started a new antihypertensive treatment, and 26 (65%) modified the previous treatment.

Among systolic parameters, only mean GLS (−22.2 ± 2.6 vs. −21.3 ± 2.5, *p* < 0.001) showed a statistically significant decrease at FU TTE; morphological parameters remained similar. No statistically significant correlation between GLS variation and cumulative dose of Carfilzomib at FU exam or BP modification was found. The percentage of diastolic dysfunction and E/e′ value increased after therapy, while LAVi and E/A ratio did not show significant changes (Table 2).

**Table 2:** Hemodynamic and echocardiographic parameters before Carfilzomib and after 6 months in the whole population.

**Population, ***n*** = 88**				
		**Baseline**	**Follow-up exam**	***P*****-value**
**Office blood pressure**				
Office SBP, mmHg		130.3 ± 18.1	124.6 ± 15.2	0.003
Office DBP, mmHg		76.3 ± 10.8	72.9 ± 9.2	0.006
BP <140/90 mmHg, *n*(%)		55 (63)	69 (78.4)	0.013
HR, bpm		75.9 ± 13.9	75.9 ± 12.8	1
Antihypertensive therapy (yes), *n*(%)		43 (48.9)	61 (69.3)	<0.001
**Echocardiography**				
LV systolic function	LVEF, %^*^	60.7 ± 5.5	59.6 ± 4.9	0.072
	GLS, %^*^	−22.2 ± 2.6	−21.3 ± 2.5	<0.001
LV morphology	LVMi, (g/m^2^)	90.6 ± 21.1	90.6 ± 23.6	0.9
	RWT	0.44 ± 0.09	0.45 ± 0.1	0.4
LV diastolic function	LAVi, ml/m^2^	28.8 ± 8.2	30.7 ± 10	0.1
	E/A	0.71 ± 0.16	0.75 ± 0.17	0.2
	E/e′	8.9 ± 2.7	9.7 ± 3.7	0.006
	Diastolic dysfunction, (%)	5 (5.6)	11 (13.4)	0.04

* *Mean values estimated on 70 patients*.

*SBP, systolic blood pressure; DBP, diastolic blood pressure; BP, blood pressure; HR, heart rate; LVEF, left ventricular ejection fraction; GLS, global longitudinal strain; LVMi, left ventricular mass indexed to body surface area; RWT, relative wall thickness; LAVi, left atrial volume indexed to body surface area; E/A, transmitral Doppler E wave velocity/transmitral Doppler A wave velocity; E/e′, transmitral Doppler E wave velocity/TDI e′ wave velocity*.

### Incidence of CVAEs During Carfilzomib Therapy and Baseline Predictors of CVAEs

**Figure 2:**
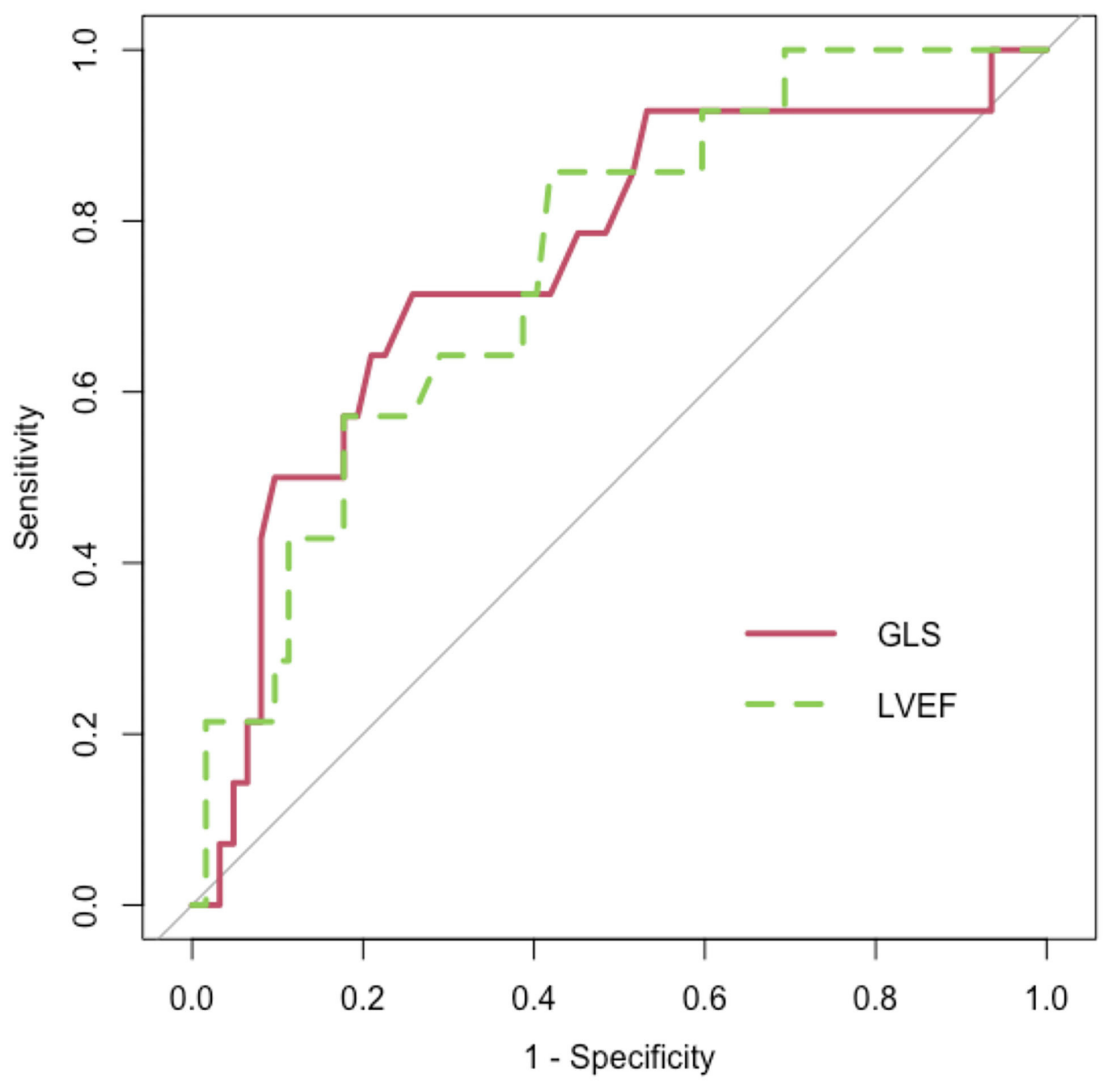
Receiver operating characteristic (ROC) curve (red) for baseline global longitudinal strain (GLS) [area under the curve (AUC) = 0.751; *p* = 0.004] and ROC curve (dashed and green) for baseline left ventricle ejection fraction (LVEF) (AUC = 0.75, *p* = 0.004).

Fifty-eight percent of the population experienced CVAEs during a median clinical follow-up of 12.4 (7.9–22.6) months, on average 3.3 (0.5–6.3) months from Carfilzomib initiation. Of the patients, 52.3% had hypertension-related CVAEs and about half of them more than one event: specifically, 43.2% experienced new onset or worsening arterial hypertension, 33% arterial hypertension just before Carfilzomib infusion and 12.5% after infusion, 4.5% uncontrolled hypertension with related symptoms, and no hypertensive emergency reported. Seventeen percent of the patients experienced major CVAEs and about a quarter of them more than one event: 4.5% had dyspnea, 5.7% arrythmia (two atrial fibrillation, one atrial bigeminy, one not-sustained ventricular tachycardia, one ventricular bigeminy/trigeminy), 4.5% severe hypotensive event, 1.1% heart failure, 3.4% typical chest pain, 3.4% patients acute coronary syndrome (one ST elevation and two non-ST elevation myocardial infarction), 1.1% syncope, and 1.1% cardiac arrest. Of the patients, 11.3% experienced both hypertensive and major CVAEs (Supplementary Table 4). There were no differences in mean age, gender, physical characteristics, and CV risk factors between the three groups that were identified based on the occurrence of hypertension-related CVAEs, major CVAEs, or no CVAEs (Table 3). At baseline, patients with no CVAEs had lower systolic and diastolic office mean BP values and a greater proportion of controlled BP. Mean baseline LVEF and GLS values significantly differed between patients with major CVAEs, hypertension-related events, and without events (LVEF, 57.1 ± 4.5% vs. 60.9 ± 4.9% vs. 62.4 ± 5.6%, *p* = 0.007; GLS, −20.3 ± 2.4% vs. −22.1 ± 2.2% vs. −22.8 ± 2.7%, *p* = 0.008, respectively), with the greatest difference between patients with major CVAEs and without events. Three patients had a baseline LVEF <50%: two of these experienced major CVAEs and one no events. Eleven patients had a GLS >–19% at the baseline TTE: six of these experienced major CVAEs, three hypertension-related events, and two no events. Mean baseline GLS and LVEF values predicted the incidence of major CVAEs during Carfilzomib therapy (*p* = 0.008 and OR = 1.407; *p* = 0.007 and OR = 1.192, respectively). Through ROC analysis, the best identified baseline GLS value that discriminated an increased risk of major CVAEs was −21.35%, with a sensitivity of 71.4% and a specificity of 74.2% (Figure 2). The best baseline LVEF value predicting an increased risk was 60.55%, with a sensitivity of 85.7% and a specificity of 58.1% (Figure 2). No statistically significative difference was detected between the area under the ROC curve (AUC) for baseline GLS and LVEF. Considering the rounded baseline values GLS ≥−21% and LVEF ≤ 60%, both predicted an increased risk of major CVAEs (*p* = 0.004 and OR = 6.2; *p* = 0.04 and OR = 3.7, respectively).

**Table 3:** General and hemodynamic baseline characteristics according to the type of cardiovascular adverse event.

**Variables**		**Population**, ***n*** **=** **88**			
		**Major CVEAs, *n* = 15**	**Hypertension-related CVAEs, *n* = 36**	**No CVAEs, *n* = 37**	***P*-value**
**General characteristics**					
Age, years		66.1 ± 5.2	65.8 ± 9.7	64.6 ± 8.9	0.8
Male sex, *n*(%)		7 (46.7)	24 (66.7)	15 (40.5)	0.07
Weight, kg		71.1 ± 15.8	74.9 ± 14.7	69 ± 14.7	0.2
Height, cm		160.4 ± 10.7	164.3 ± 11.5	159.7 ± 9.5	0.2
BSA, m^2^		1.7 ± 0.2	1.8 ± 0.2	1.7 ± 0.2	0.1
BMI, kg/m^2^		27.4 ± 4.1	27.7 ± 4.5	27 ± 4.6	0.8
**CV risk factors**					
Active smoke, *n*(%)		1 (6.7)	2 (5.6)	3 (8.1)	0.8
Arterial hypertension, *n*(%)		8 (53.3)	20 (55.6)	16 (43.2)	0.6
Obesity, *n*(%)		4 (26.7)	9 (25)	13 (35.1)	0.6
Coronary heart disease, *n*(%)		2 (13.3)	0 (0)	1 (2.7)	0.06
Diabetes mellitus, *n*(%)		1 (6.7)	5 (13.9)	4 (10.8)	0.8
Chronic renal failure, *n*(%)		3 (20)	1 (2.8)	5 (13.5)	0.1
Dyslipidemia, *n*(%)		1 (6.7)	7 (19.4)	6 (16.2)	0.5
**Office blood pressure**					
Office SBP, mmHg		134.9 ± 20.7	134.6 ± 15.3^#^	124.2 ± 18.3^#^	0.03
Office DBP, mmHg		77.1 ± 6.8	79.9 ± 10.1^#^	72.2 ± 12.1^#^	0.009
BP <140/90 mmHg, *n*(%)		10 (66.7)	17 (47.2)^#^	28 (75.7)^#^	0.04
HR, bpm		74.4 ± 14	76.1 ± 13	76 ± 14	0.9
Antihypertensive therapy (yes), *n*(%)		9 (60)	16 (44.4)	18 (48.6)	0.6
**Baseline echocardiography**					
LV systolic function	LVEF, %^*^	57.1 ± 4.5^∧^	60.9 ± 4.9	62.4 ± 5.6^∧^	0.007
	GLS, %^*^	−20.3 ± 2.4^∧^	−22.1 ± 2.2	−22.8 ± 2.7^∧^	0.008
LV morphology	LVMi, (g/m^2^)	87.7 ± 22	96.9 ± 22.3	88.4 ± 19.8	0.2
	RWT	0.45 ± 0.09	0.45 ± 0.1	0.42 ± 0.06	0.1
LV diastolic function	LAVi, ml/m^2^	28.2 ± 7.7	27.5 ± 8.6	29.7 ± 8.3	0.5
	E/A	0.92 ± 0.22	0.92 ± 0.3	1 ± 0.28	0.1
	E/e′	8.9 ± 2.9	8.8 ± 2.3	9.3 ± 2.6	0.7

# *p < 0.05 between groups 2 and 3*.

∧ *p < 0.05 between groups 1 and 3*.

* *Mean values estimated on 70 patients (group 1, n = 14; group 2, n = 30, group 3, n = 32)*.

*BSA, body surface area; BMI, body mass index; MM, multiple myeloma; SBP, systolic blood pressure; DBP, diastolic blood pressure; BP, blood pressure; HR, heart rate; LVEF, left ventricular ejection fraction; GLS, global longitudinal strain; LVMi, left ventricular mass indexed to body surface area; RWT, relative wall thickness; LAVi, left atrial volume indexed to body surface area; E/A, transmitral Doppler E wave velocity/transmitral Doppler A wave velocity; E/e′, transmitral Doppler E wave velocity/TDI e′ wave velocity*.

### Hemodynamic and Echocardiographic Variation After Carfilzomib in CVAE-Related Groups

After 6 months of therapy, we observed a decreasing trend of BP values in all CVAE-related groups (Supplementary Table 5). Subsequently, we analyzed the echocardiographic parameters modifications (Table 4). Patients with major CVAEs showed similar systo-diastolic parameters on TTE before and after therapy. A statistically significant worsening in mean GLS was observed both in patients with hypertensive events and without CVAEs. LVEF was slightly reduced in patients with no adverse events.

**Table 4:** Echocardiographic parameters before Carfilzomib and 6 months follow-up evaluation according to the type of cardiovascular adverse event (CVAE).

**TTE**		**Major CVAEs**, ***n*** **=** **15**			**Hypertension-related CVAEs**, ***n*** **=** **36**			**No CVAEs**, ***n*** **=** **37**		
		**Baseline**	**FU exam**	***P*-value**	**Baseline**	**FU exam**	***P*-value**	**Baseline**	**FU exam**	***P*-value**
LV systolic function	LVEF, %^*^	56.9 ± 4.9	58.5 ± 4.9	0.15	60.7 ± 5	59 ± 5.4	0.15	62.3 ± 5.5	60.6 ± 4.3	0.044
	ΔLVEF^*^	+1.7 ± 3.7			−1.7 ± 5.9			−1.7 ± 4.4		0.09
	GLS, %^*^	−20.4 ± 2.6	−20.7 ± 3.3	0.5	−22 ± 2.3	−21 ± 2.3	0.002	−23.1 ± 2.5	−21.7 ± 2.3	<0.001
	ΔGLS^*^	−0.35 ± 1.7^#^			+1 ± 1.6			+1.4 ± 1.8^#^		0.01
LV morphology	LVMi, (g/m^2^)	86.2 ± 22	94.4 ± 32	0.25	95.7 ± 21.7	92.3 ± 18.9	0.4	87.9 ± 19.7	87.6 ± 23.9	0.9
	RWT	0.45 ± 0.09	0.47 ± 0.08	0.5	0.46 ± 0.1	0.45 ± 0.09	0.8	0.41 ± 0.07	0.44 ± 0.1	0.2
LV diastolic function	LAVi, ml/m^2^	28.6 ± 7.9	33.1 ± 12.6	0.15	27.3 ± 8.6	31 ± 9.9	0.052	30.4 ± 7.8	29.3 ± 9	0.6
	E/A	0.91 ± 0.22	0.86 ± 0.12	0.2	0.93 ± 0.31	0.92 ± 0.31	0.9	1 ± 0.28	0.96 ± 0.22	0.09
	E/e′	8.9 ± 2.9	10 ± 5.1	0.4	8.8 ± 2.2	10.3 ± 3.5	0.006	9.3 ± 2.6	9.8 ± 2.3	0.1
	Diastolic dysfunction, *n*(%)	1 (6.6)	3 (20)	0.5	3 (8.3)	6 (16.6)	0.1	1 (2.7)	2 (5.4)	1

* *Mean values estimated on 70 patients (group 1, n = 12; group 2, n = 28; group 3, n = 30)*.

# *P < 0.05 between groups 1 and 3*.

*FU, follow-up; TTE, transthoracic echocardiography; LVEF, left ventricular ejection fraction; GLS, global longitudinal strain; LVMi, left ventricular mass indexed to body surface area; RWT, relative wall thickness; LAVi, left atrial volume indexed to body surface area; E/A, transmitral Doppler E wave velocity/transmitral Doppler A wave velocity; E/e′, transmitral Doppler E wave velocity/TDI e′ wave velocity*.

Comparisons among groups highlighted a significant difference in terms of GLS variation (ΔGLS) during Carfilzomib between patients with major CVAEs and patients without events, while LVEF variation (ΔLVEF) did not differ between groups. No variation in terms of LV morphology (LVMi and RWT) was detected. No statistically significant variation in diastolic parameters was found after Carfilzomib treatment, except for a significant increase in E/e′ values in patients with hypertensive events (Table 4).

## Discussion

Carfilzomib seems to cause a mild subclinical impairment of LV systo-diastolic function assessed by TTE. In addition, baseline LVEF and GLS may have a predictive role in identifying patients with an increased risk of CVAEs during treatment.

The efficacy of Carfilzomib in RRMM is well-established, but the antiproteasome activity may have adverse consequences on cardiovascular system. Arterial hypertension is well recognized as one of the most frequent CVAEs (2–5, 35, 36); moreover, arterial hypertension should be treated before starting Carfilzomib infusions because it is an important predictor of CVAEs (37, 38). In our population, the optimization of antihypertensive treatment led to a better BP control after 6 months. Nevertheless, despite this improved control and specific follow-up, 52.3% of patients still experienced hypertensive CVAEs, suggesting that optimization of BP values alone could not eliminate the risk of arterial-hypertension-related CVAEs.

At the present time, the predictive and prognostic values of echocardiographic monitoring in patients receiving Carfilzomib are still under investigation, with limited evidences (4, 13, 39, 40). The position statement of the Heart Failure Association, the European Association of Cardiovascular Imaging, and the Cardio-Oncology Council of the European Society of Cardiology (41) suggests an echocardiographic surveillance in medium-/high-risk patients receiving Carfilzomib and strongly recommends prompt echocardiography in the presence of new cardiac signs/symptoms. Our study showed that patients treated with Carfilzomib have early (after five to six cycles) but minimal worsening in LV systolic function assessed by GLS (−22.2 ± 2.6 vs. −21.3 ± 2.5, *p* < 0.001), in line with previously reported data (42). However, this negative effect on LV systolic function is not predictive of a subsequent clinically relevant cardiac dysfunction. We analyzed echocardiographic changes occurring within few months from the start of therapy (6 months), while CVAEs were recorded over the entire therapy duration (which varies according to the individual hematological indication). It may be possible that cardiac mechanic alterations potentially related to CVAEs were not evident after just 6 months of Carfilzomib treatment, and we could suppose that cardiac alteration that leads to major CVAEs may be evident after more than 6 months of therapy. Moreover, CVAEs incidence may not be entirely explained by cardiac function variations: endothelial dysfunction caused by PI may play an important pathogenetic role (13).

Anyway, worsening of GLS suggests a subclinical damaging effect of Carfilzomib on LV function. Diastolic dysfunction has been explored as a marker of early cardiotoxicity, but the current evidence does not support its role for the prediction of later cardiac dysfunction (43). In our cohort, Carfilzomib showed a detrimental effect on diastolic function: prevalence of diastolic dysfunction in our study population rose from 5.6 to 13.4%, and mean value of E/e′ increased (Table 2). This result is in accordance with previous data that showed a diastolic function change after four cycles of Carfilzomib (44).

In our cohort, 58% of patients experienced CVAEs: 41% experienced only hypertensive CVAEs, while 17% experienced major CVAEs. Patients in the latter group did not show a decrease in functional echocardiographic parameters at the 6 months evaluation, maybe for a limited functional reserve in presence of compromised baseline functional parameters. Hence, in our cohort, a GLS impairment after 6 months of Carfilzomib was not a predictor of CVAEs, while baseline GLS ≥−21% and LVEF ≤ 60% represented predictors of major CVAEs during therapy. We could suppose that Carfilzomib mediates a greater cardiovascular damage, which became clinically evident with the occurrence of CVAEs, in patients with worse functional baseline parameters before beginning a potentially cardiotoxic drug. The echocardiographic baseline functional evaluation could identify the group of patients with an increased risk of major CVAEs if exposed to Carfilzomib treatment, and therefore, it has a relevant prognostic value in MM patients treated with Carfilzomib, in order to identify patients at risk of CV complications.

Our study has some limitations. We have a relatively small cohort, and the GLS analysis with the dedicated software was feasible in 80% of our patients. Observed incidence of arterial hypertension after Carfilzomib may be limited by the BP optimization at the baseline, but, at the same time, we observed a greater hypertension rate than previously reported in the literature, probably because of our attention as a specialized center (Hypertension Unit). Moreover, FU period was limited by therapy duration; further studies are needed to determine the incidence of long-term CVAEs after Carfilzomib conclusion.

## Conclusions

In conclusion, our study suggests that Carfilzomib causes an early mild LV systolic function impairment, demonstrated by GLS change after few months of treatment, as well as increases diastolic dysfunction. These functional echocardiographic variations did not directly translate into higher incidence of CVAEs during therapy, reflecting only a subclinical effect on cardiac mechanic. However, baseline echocardiographic parameters may predict the incidence of CVAEs during therapy, and therefore, the echocardiographic baseline assessment plays an essential role in assessing the cardiovascular risk of patients with clinical indication to Carfilzomib. The presence of one among GLS ≥−21% and LVEF ≤ 60% before therapy initiation may identify patients with an increased risk of experiencing CVAEs during treatment. For these patients, an early initiation of appropriate cardioprotective measures and monitoring might be warranted in order to reduce the incidence of CVAEs and the subsequent therapy withdrawal.

## Data Availability Statement

The original contributions generated for the study are included in the article/Supplementary Material, further inquiries can be directed to the corresponding author/s.

## Ethics Statement

The studies involving human participants were reviewed and approved by A.O.U. Città della Salute e della Scienza of Turin (Protocol Number 0038655). The patients/participants provided their written informed consent to participate in this study.

## Author Contributions

GM wrote the manuscript. AA, AM, and LA contributed to the data analysis. AM, FVa, GB, DL, EA, and FVe revised the manuscript. GC, MS, SB, and FG contributed for the hematologic data and revised the manuscript. All authors contributed to the data collection.

## Conflict of Interest

The authors declare that the research was conducted in the absence of any commercial or financial relationships that could be construed as a potential conflict of interest.

## Abbreviations

MM: multiple myeloma
PI: proteasome inhibitor
RRMM: relapsed or refractory multiple myeloma
LV: left ventricle
LVEF: left ventricle ejection fraction
GLS: global longitudinal strain
2D-STE: 2D speckle-tracking echocardiography
CV: cardiovascular
CVAE: cardiovascular adverse event
BP: blood pressure
TTE: transthoracic echocardiography
ICC: interclass coefficient correlations
CI: confidence interval
FU: follow-up
LVMi: left ventricular mass indexed to body surface area
RWT: relative wall thickness
LAVi: left atrial volume indexed to body surface area
TDI: tissue Doppler imaging
E/A: transmitral Doppler E wave velocity/transmitral Doppler A wave velocity; E/e′ = transmitral Doppler E wave velocity/TDI e′ wave velocity.
