# Supplementary material for: Effects of Carfilzomib Therapy on Left Ventricular Function in Multiple Myeloma Patients
Source: Front Cardiovasc Med. 2021 Apr 21;8:645678. doi: 10.3389/fcvm.2021.645678 (PMC8096903; doi:10.3389/fcvm.2021.645678)
Supplement: Supplementary file 1 [file Data_Sheet_2.docx]

Supplementary Material

| Protocol | Chemotherapeutic agents | Administration days  (Cycles of 28 days) | Number of Patients* |
| --- | --- | --- | --- |
| KD | - Carfilzomib 56mg/m^2^ (20mg/m^2^ D1 and D2 of C1)  - Dexamethasone 20 mg | - D1, 2, 8, 9, 15, 16  - D1, 2, 8, 9, 15, 16, 22, 23 | **22** |
| KRD | - Carfilzomib 27 mg/ m^2^ (20mg/m^2^ D1 and D2 of C1)  - Dexamethasone 40 mg  - Lenalidomide 25 mg | - D1, 2, 8, 9, 15, 16  (from C13: 1, 2, 15, 16)  - D1, 8, 15, 22  - From D1 to D21 | **41** |
| FORTE  Phase III study in newly diagnosed MM | **Study arm 1 (ASCT after 4 cycles):**  - Carfilzomib 36 mg/m^2^ (20mg D1 and D2 of C1)  - Dexamethasone 40 mg  - Lenalidomide 25 mg | - D1, 2, 8, 9, 15, 16  - D1, 2, 8, 9, 15, 16, 22, 23  - From D1 to D21 | **4** |
|  | **Study arm 2:**  - Carfilzomib 36 mg/m^2^ (20mg D1 and D2 of C1)  - Dexamethasone 40 mg  - Lenalidomide 25 mg | - D1, 2, 8, 9, 15, 16  - D1, 2, 8, 9, 15, 16, 22, 23  - From D1 to D21 |  |
|  | **Study arm 3 (ASCT after 4 cycles):**  - Carfilzomib 36 mg/m^2^ (20mg D1 and D2 of C1)  - Dexamethasone 20 mg  - Cyclophosphamide 300 mg/m^2^ | - D1, 2, 8, 9, 15, 16  - D1, 2, 8, 9, 15, 16, 22, 23  - D1, 8,15 |  |
| EMN07  Phase I/II study in RRMM | - Carfilzomib 27/36/45/56 mg/m^2^  (level -1/0/+1/+2) (20mg/m^2^ D1 of C1)  - Dexamethasone 20 mg  - Pomalidomide 4 mg | - D1, 8, 15  - D1, 8, 15, 22  - From D1 to 21 | **11** |
| ARROW  Phase III study in  RRMM | **Study arm A:**  - Carfilzomib 70 mg/m^2^ (20mg/m^2^ D1 of C1)  - Dexamethasone 40 mg | - D1, 8, 15  - D1, 8, 15 (22 from C1 to C9) | **2** |
|  | **Study arm B:**  - Carfilzomib 27 mg/m^2^ (20mg/m^2^ D1 and D2 of C1)  - Dexamethasone 40 mg | - D1, 2, 8, 9, 15, 16  - D1, 8, 15 (22 from C1 to C9) | **2** |
| EMN11  Phase I/II study in  RRMM | **(ASCT after 4 cycles)**  - Carfilzomib 36 mg/m^2^ (20mg/m^2^ D1-D2 of C1)  - Dexamethasone 20 mg  - Pomalidomide 4 mg | - D1, 2, 8, 9, 15, 16  - D1, 2, 8, 9, 15, 16  - From D1 to D21 | **3** |

**Table S1. Carfilzomib-based chemotherapeutic protocols.**

*1 patient underwent EFC15246 (Isatuximab- Carfilzomib- Dexamethasone) phase III study protocol, 2 patients had unknown protocol.

D: days; C: cycle; MM: multiple myeloma; RRMM: refractory/relapsed multiple myeloma, ASCT: autologous stem cell transplant.

**Table S2. Timing and cumulative dose of Carfilzomib at the 6-month follow-up (FU) echocardiogram (TTE), at the time of the first cardiovascular adverse event (CVAE) and at the end of planned therapy.**

|  | | | | |
| --- | --- | --- | --- | --- |
|  | **FU TTE**  *(n=88)* | **Hypertension-related CVAEs** *(n=46)* | **Major CVAEs**  *(n=15)* | **End of therapy**  *(n=88)* |
| Time,  *months* | 5.4  [4.3-6.4] | 3.6  [0.5-7.6] | 5.4  [0.9-7] | 10.4  [6.7-18.7] |
| Carfilzomib,  *mg* | 1413.4  [1140.4–2105.2] | 1169.9  [99.6 - 2330.4] | 1750.3  [953-3232.8] | 3193  [1494.1-5319.3] |

**Table S3. Interclass correlation coefficients (ICC) for GLS and left ventricular function (LVEF) assessments on 10 measurements.**

|  |  |  |  |
| --- | --- | --- | --- |
| Parameter | **Type of comparison** | **ICC** | **CI** |
| GLS | Inter-operator, *%* | 97 | 91-99 |
|  | Intra-operator, *%* | 98 | 94-99 |
| LVEF | Inter-operator, *%* | 94 | 75-98 |
|  | Intra-operator, *%* | 94 | 77-98 |

ICC: interclass correlation coefficients; CI: confidence interval.

ICC values less than 50% are indicative of poor reliability, values between 50% and 75% indicate moderate reliability, values between 75% and 90% indicate good reliability, and values greater than 90% indicate excellent reliability.

**Table S4. Cardiovascular adverse events (CVAEs) incidence during Carfilzomib therapy.**

|  | |
| --- | --- |
| **Cardiovascular adverse events*** | **Population,** *n*=88 |
| **Events related to arterial hypertension, *(%)*** | 46 (52.3) |
| Arterial hypertension, *(%)* | 38 (43.2) |
| Arterial hypertension before Carfilzomib infusion, *(%)*  -with subsequent administration, *(%)*  - without subsequent administration, *(%)* | 29 (33)  19 (21.6)  10 (11.4) |
| Arterial hypertension after Carfilzomib infusion, *(%)* | 11 (12.5) |
| Uncontrolled arterial hypertension (>180/100)  with symptoms, *(%)* | 4 (4.5) |
| Hypertensive emergency*, (%)* | 0 (0) |
| More than 1 hypertensive event, *(%)* | 25 (54.3) |
| **Major cardiovascular events, *(%)*** | 15 (17) |
| Dyspnea, *(%)* | 4 (4.5) |
| Arrythmia, *(%)* | 5 (5.7) |
| Severe hypotension, *(%)* | 4 (4.5) |
| Cardiac failure, *(%)* | 1 (1.1) |
| Typical chest pain, *(%)* | 3 (3.4) |
| STEMI, *(%)* | 1 (1.1) |
| NSTEMI, *(%)* | 2 (2.2) |
| Syncope, *(%)* | 1 (1.1) |
| Cardiac-related sudden death, *(%)* | 1 (1.1) |
| More than 1 major CVAEs, *(%)* | 4 (26.6) |
| **Both major and hypertensive events, *(%)*** | 10 (11.3) |

*Defined according to CTCAE 5.0 (Common Terminology Criteria for Adverse Events).

**Table S5. Office blood pressure (BP) values before and after 6 months of Carfilzomib therapy, according to the type of cardiovascular adverse event (CVAE).**

|  | | | | | | | | | |
| --- | --- | --- | --- | --- | --- | --- | --- | --- | --- |
| Office BP  values | **Major CVAEs**  *n*=15 | | | **Hypertension-related CVAEs**  *n*= *36* | | | **No CVAEs**  *n*=37 | | |
|  | Baseline | FU exam | p-value | Baseline | FU exam | p-value | Baseline | FU exam | p-value |
| SBP, *mmHg* | 134.9 ± 20.7 | 124.7 ± 18.2 | 0.09 | 134.4 ± 15.5 | 130 ± 16.2 | 0.2 | 124.7 ± 18.3 | 119.3 ± 11.7 | 0.04 |
| ∆ SBP, *mmHg* | 4.5 [-9.5; 31.3] | | | 5 [-8; 17] | | | 2.5 [-5; 14.8] | | 0.3 |
| DBP, *mmHg* | 77.1 ± 6.8 | 72.3 ± 9.1 | 0.07 | 79.7 ± 10.2 | 74.5 ± 17 | 0.01 | 72.7 ± 11.8 | 71.6 ± 8.9 | 0.5 |
| ∆ DBP, *mmHg* | 4.9 ± 9.2 | | | 5.1 ± 11.3 | | | 1.1 ± 1.6 | | 0.3 |
| MBP, *mmHg* | 96.4 ± 10 | 89.8 ± 11.3 | 0.059 | 97.9 ± 10.5 | 93.7 ± 12.3 | 0.07 | 90 ± 12.6 | 87.5 ±7.8 | 0.2 |
| ∆MBP, *mmHg* | 6.6 ± 11 | | | 4.2 ± 13.7 | | | 2.6 ± 11.4 | | 0.5 |
| DiBP, *mmHg* | 57.7 ± 18.1 | 52.4 ± 13.1 | 0.2 | 54.7 ± 13.1 | 55.5 ± 13.2 | 0.7 | 52 ± 14.3 | 47.8 ± 13.2 | 0.04 |
| ∆DiBP, *mmHg* | 5.3 ± 16.3 | | | -0.77 ± 11.8 | | | 4.3 ± 12.1 | | 0.2 |

FU: follow-up; SBP: systolic blood pressure, DBP: diastolic blood pressure, MBP: mean blood pressure, DiBP: differential blood pressure.
